# mRNA-based SARS-CoV-2 Comirnaty vaccine elicits weak and short specific memory B cell response in individuals with no previous infection

**DOI:** 10.3389/fimmu.2023.1127379

**Published:** 2023-06-29

**Authors:** José L. Casado, Pilar Vizcarra, Adrián Martín-Hondarza, Sandra Gómez-Maldonado, Magdalena Muedra-Sánchez, Judith del Pino, Itria G. Mirabella, Sara Martín-Colmenarejo, Johannes Haemmerle, Marina Fernández-Escribano, Alejandro Vallejo

**Affiliations:** ^1^ Department of Infectious Diseases, CIBERINFEC (Biomedical Research Center Network in Infectious Diseases), Madrid, Spain; ^2^ IRYCIS, Instituto Ramón y Cajal de Investigaciones Sanitarias, University Hospital Ramón y Cajal, Madrid, Spain; ^3^ Laboratory of Immunovirology, University Hospital Ramón y Cajal, Madrid, Spain; ^4^ Department of Occupational Safety and Health, University Hospital Ramón y Cajal, Madrid, Spain

**Keywords:** SARS-CoV-2, convalescents, COVID-19, longterm immunity, B cell response

## Abstract

**Objectives:**

The dynamics of the memory B cell (MBC) repertoire after SARS-CoV-2 vaccination is crucial for assessing long-term immunity. We compare spike-specific MBC responses between SARS-CoV-2 unexposed and recovered individuals, and their impact on breakthrough infections during follow-up.

**Methods:**

Spike-specific MBC and T cells were quantified at inclusion and after two doses of mRNA vaccine in a longitudinal cohort of 85 naïve and 64 recovered participants (47 with positive serology and 17 with negative serology after infection).

**Results:**

At inclusion, there was minimal spike-specific MBC in naïve SARS-CoV-2 individuals. After the second vaccine dose, MBCs were significantly boosted in naïve individuals, but reached a significantly lower level than that observed even in unvaccinated SARS-CoV-2 convalescents (p<0.001). Furthermore, while the secondary memory B cell (MBC) population consisted of 100%, 33%, and 76% IgG^+^, IgM^+^, and IgA^+^ expressing cells, respectively, in the unexposed group, the MBC response showed a significant decrease across all isotypes. Similarly, although secondary specific IgG^+^, IgM^+^, and IgA^+^-MBC isotypes were found in 100%, 39%, and 76% of the unexposed participants, respectively, the magnitude of the MBC levels was significantly lower for all the isotypes compared to convalescents. Interestingly, convalescents without an initial serological response had a lower MBC response, like what found in unexposed subjects. There was an inverse correlation between specific MBCs (r=-0.307; p=0.027), especially for isotype IgA^+^ (r=-0.279, p=0.045), and the time since the second vaccination dose. Furthermore, during a median follow-up of 434 days (IQR, 339-495), 49 out of 149 individuals (33%) became infected, 29 in naïve and 20 in convalescent individuals, showing a significant correlation between spike-specific MBC magnitude after vaccination and the time for SARS-CoV-2 infection, especially for IgA^+^/IgG^+^ MBC isotypes.

**Conclusions:**

MBCs were primed by mRNA-based vaccination in most cases, but SARS-CoV-2 naïve individuals had a blunted specific MBC response, and this was associated with a shorter time to breakthrough SARS-CoV-2 infection.

## Introduction

The introduction of vaccines has resulted in the prevention of SARS-CoV-2 infection and the management of disease severity (1, 2). However, there are conflicting results regarding the longevity of antibody response to SARS-CoV-2 (3), and, in addition, studies in vaccinated individuals have mainly focused on the serological response and neutralising antibodies (4).

The immune response to infection or vaccination results in the production of antibodies by antibody-secreting cells (ASC), which can provide rapid serological immunity and the generation of long-lived MBC (5). MBC can persist for decades or potentially for life, in lymph nodes, spleen, bone marrow and lungs, or circulate in the blood (6). For example, after vaccination, COVID-19-recovered patients showed a striking expansion of spike-specific MBCs (7), but less is known about the long-term dynamics of the MBC repertoire after vaccination in unexposed individuals despite repeated antigenic stimulation. Indeed, there are data, suggesting that long-lived plasma cells may not have been efficiently generated following vaccination regimens (8).

Here, we studied two longitudinal cohorts of naïve individuals and recovered patients for up to 2 months after two doses of the mRNA-based BNT162b2 (Pfizer BioNTech) COVID-19 vaccine to understand how mRNA vaccination impacts the MBC pool shaped by previous exposure to SARS-CoV-2 and to decipher how MBCs from naïve vaccinees differ and evolve compared to SARS-CoV-2 recovered patients.

## Methods

This study included 149 healthcare workers, 64 COVID-19 convalescents, and 85 infection-naïve individuals who were followed at the tertiary Ramon y Cajal University Hospital (Madrid, Spain) since March 2020. They were over 18 years of age, without immunodeficiency or immunosuppression, and without cancer or immunosuppressive treatment. They had participated in an internal survey for the presence of antibodies against the SARS-CoV-2 N protein after the first wave of the disease (9), and after the inclusion in the study. They were then vaccinated with two doses of the mRNA-based BNT162b2 (Pfizer BioNTech) COVID-19 vaccine in January-February 2021. Among them, 38 naïve, 8 convalescents without initial positive serology and 16 convalescents with initial positive serology, participated in the blood sampling and were analysed. Thus, three time-points were analyzed: an internal serological survey (April 2020), inclusion into the study (October 2020), and 3-4 weeks after the second dose of mRNA vaccine (February 2021). This study design allowed us to investigate the kinetics of immune responses following infection and after vaccination.

Convalescent patients were defined as those with suggestive symptoms and a positive nasopharyngeal swab PCR positive test for SARS-CoV-2. This group was subdivided according to the serological response in the initial serological survey, as measured by anti-N IgG antibodies (COVID-19 IgG/IgM Rapid Test Kit, UNscience Biotechnology, Wuhan, China; and COVID-19-SARS-CoV-2 IgA ELISA, Demeditech, Germany) since 17 out of 64 convalescent patients had a positive nasopharyngeal swab RT-PCR test with repeated negative serology since the first days after the infection. The infection-naïve (unexposed) group were those with no suggestive symptoms, negative SARS-CoV-2-specific PCR (if performed), and no presence of anti-N IgG antibodies at the internal survey and the inclusion time points. Among convalescents, mild disease was defined as the presence of symptoms attributable to COVID-19 in absence of radiological infiltrates and lack of hypoxaemia (oxygen saturation ≥95% on room air). Moderate disease was defined as the presence of radiological infiltrates with oxygen saturation ≥95% on room air. Severe disease was defined as the presence of any of the following: oxygen saturation ≤93% at rest; arterial partial pressure of oxygen (PaO2)/fraction of inspired oxygen (FiO2) ≤300 mmHg (1 mmHg = 0.133 kPa) (10). Due to the bias of HCWs being attended at home even in the case of more severe disease (or the bias of possible admission for better attention to colleagues), hospitalisation was not considered as severe disease in the absence of other criteria.

Approximately one year after vaccination (median time of follow-up of 434 days), information on confirmed breakthrough infections was obtained and recorded from all the participants (N=149). These infections were confirmed by positive PCR or antigen detection from nasopharyngeal swabs. This information was recorded prior to receiving the booster dose (third dose) of Pfizer COVID-19 bivalent (Comirnaty original/Omicron BA.4-5) vaccine (November 2022).

All patients gave written informed consent at inclusion, and the study was approved by the institutional review boards of our Hospital Ethics Committee (EC162/20) and registered at the clinical trials repository (clinicaltrials.gov, NCT04402827).

### Laboratory analysis

Peripheral blood mononuclear cells (PBMC) were isolated from EDTA blood samples by Ficoll-Paque density gradient centrifugation using lymphocyte separation medium (Corning, New York, NY) and cryopreserved. Plasma samples were stored at -80°C.

All the participants (naïve and convalescent) were tested for anti-N SARS-CoV-2 IgG antibodies (COVID-19-SARS-CoV-2 IgG ELISA, Demeditech, Germany) at inclusion and after a median of 17 days after the second vaccine dose to confirm serological status independent of the antibody production to the vaccine. Results were expressed as relative units per milliliter (U/mL), with a cut-off of 11 U/mL. They were also tested for anti-Spike IgG antibodies (SARS-CoV-2 IgG II Quant, Abbott, Maidenhead, United Kingdom) with a cut-off of 50 arbitrary units per milliliter (AU/mL) and for both anti-S IgA and anti-S IgM (COVID-19-SARS-CoV-2 IgA ELISA, COVID-19-SARS-CoV-2 IgM ELISA Demeditech, Germany), with a threshold of 0.1 U/mL at baseline and after both doses. All these assays used the SARS-CoV-2 B.1.351 lineage (beta variant) for antibody detection.

### Determination of SARS-CoV-2 neutralising antibody

SARS-CoV-2 neutralising antibodies were quantified using plasma samples from the participants and using the competitive inhibition enzyme immunoassay technique (Human Novel Coronavirus (SARS-CoV-2) Neutralising Antibody ELISA Kit, MyBioSource) according to the manufacturer´s instructions. Plate wells are precoated with SARS-CoV-2 RBD (B.1.351 lineage, beta variant) and horseradish peroxidase-conjugated ACE2 is added with the sample. The competitive inhibition reaction between HRP-ACE2 and SARS-CoV-2 neutralizing antibodies in samples is initiated. A substrate solution is added to the wells and the colour develops opposite to the amount of SARS-CoV-2 neutralising antibody present in the sample. Optical densities greater than half the optical density for the plasma-free well were considered negative. Results were expressed as ng/mL.

### Determination of SARS-CoV-2 Spike-specific memory B cells

**Figure 1:**
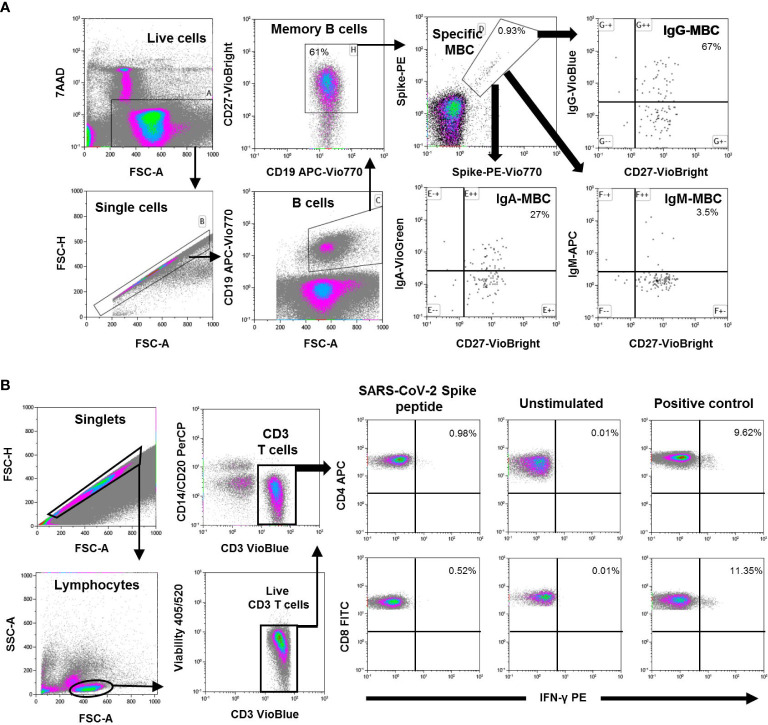
**(A)** Flow cytometry strategy for the quantification of SARS-CoV-2-specific memory B cells (MBC) from peripheral mononuclear cells (PBMC) of one representative individual. Viable cells (FSC-A/7AAA plot) were plotted with FSC-H/FSC-A parameters to exclude doublets. Single cells were then gated using CD19-APC-Vio770 and CD27-Vio-Bright-FITC to identify memory B cells. Spike-specific B cells were then identify with a double staining with the two spike-tetramer on the diagonal of the dot plot. Finally, the use of IgG-VioBlue, IgA-VioGreen, and IgM-APC were used to quantify each specific isotype of spike-specific memory B cells. Results are recorded as percentage among total memory B cells and isotypes among specific memory B cells. **(B)** Flow cytometry gating strategy for the quantification of SARS-CoV-2 spike specific T cells from PBMCs in one representative individual. After gating of singlet cells (FSC-A/FSC-H density plot), lymphocytes were morphologically selected with FSC-A/SSC-A density plot, and then CD3^+^ T cells were gated. Cell debris, monocytes, and B cells were excluded from the analysis with CD14- and CD20-PerCP antibodies, and live CD3^+^ T cells were selected. IFN-γ expression was finally analyzed separately for CD4^+^ and CD8^+^ T cells and analyzed under three different conditions; Stimulated with SARS-CoV-2 spike peptides, unstimulated (negative control), and SEB-stimulated (positive control). Results are shown as percentage of cells expressing IFN-γ.

SARS-CoV-2-specific MBC detection was performed by binding the recombinant spike protein to the corresponding antigen-specific B cell receptor (BCR) on circulating B cells (SARS-CoV-2 spike B cell analysis kit, Miltenyi Biotec, Germany) by multiparametric flow cytometry (**Figure 1A** for cytometry strategy). Tetramers formed from recombinant SARS-CoV-2 Spike-Prot (HEK)-Biotin with streptavidin, PE and PE-Vio770, respectively, were used according to the manufacturer´s instructions. The SARS-CoV-2 RBD B.1.1.7 (HEK) protein covers amino acids R319 to S591 of the spike protein, contains the N501Y mutation, as well as a C-terminal His-tag and an N-terminal AviTag. This quantitative and qualitative analysis of specific MBC and isotypes IgG+, IgM+, and IgA+, was performed by single-cell flow cytometry using fluorochrome-conjugated antibodies, and the 7-AAD to exclude of dead and apoptotic cells using a minimum of 5x10^6^ PBMCs for each analysis. Results are expressed as percentages of total memory B cells and isotypes of specific MBCs.

### Determination of SARS-CoV-2 Spike-specific T cells

Specific CD4^+^ and CD8^+^ T cell responses were performed by intracellular cytokine staining using multiparametric flow cytometry (Rapid Cytokine Inspector CD4/CD8 T cell kit, Miltenyi, Germany) in fresh blood samples. Briefly, SARS-CoV-2-specific T cells were measured using *in vitro* stimulation with SARS-CoV-2 peptide pools (SARS-CoV-2 B.1.351 lineage, beta variant) of viral proteins encompassing the spike protein followed by quantification of CD4^+^ and CD8^+^ T cell-specific interferon (IFN)-γ, using peripheral blood mononuclear cell (PBMC) samples from all subjects. A result 2-fold higher than the negative control (unstimulated) was considered positive. The complete flow cytometry strategy is shown in **Figure 1B**.

### Statistical analysis

Continuous variables were expressed as the median and interquartile range (IQ_25-75_) and categorical variables by frequencies and proportions. The Mann-Whitney *U* test (non-parametric) for independent samples was used to compare continuous variables. The Wilcoxon signed-rank test was used to compare paired samples to analyse the evolution of the measures after vaccination. Spearman’s rank correlation coefficient was used to measure the association between two variables. Differences between categorical variables were assessed using contingency tables (Chi-square distribution).

## Results

We included 149 healthcare workers, including 85 individuals who had no history of COVID-19 disease, as confirmed by negative PCR (if performed) and anti-N negative serology at inclusion. As a control, we included a cohort of 64 convalescent individuals (with the presence of symptoms and positive PCR test), with (N=47) or without (N=17) previous serological response. Demographic and baseline characteristics are described in **Table 1**. As shown, the two cohorts were similar in terms of age, sex, and body mass index. In the convalescent cohort, SARS-CoV-2 infection had occurred a median time of 185 days prior to immune assessment (inclusion). However, the group of 17 convalescents who did not have initial positive serology at the time of the survey were younger.

**Table 1:** Baseline characteristics of the individuals included in this study.

	Infection-naïve (n=85)	Convalescents (n=64)	P
Age (years)	46 (25-66)	41 (26-67)	0.184
Sex (Female)	61 (72)	41 (65)	0.36
Body Mass Index	23.2 (21.2-26.3)	23.6 (22-26.8)	0.392
Obesity (>30)	7 (8)	8 (12)	
Smoking	22 (26)	25 (39)	0.005
Diabetes mellitus	4 (5)	0	0.098
Hypertension	6 (7)	6 (9)	0.781
Days from COVID-19 to inclusion	–	198 (179-216)	–
COVID-19 illness			
Mild	–	51	
Moderate	–	4	
Severe	–	9	
Serology at study inclusion			
Anti-N IgG positive	0	37 (58)	<0.001
IgG titer (AU/ml)	4.62 (3.7-6.2)	10.1 (5.3-19.8)	<0.001
Neutralysing antibodies (ng/mL)	0	1692 (1004-2562)	
T-cell response to Spike antigen			
CD4+ T cells	35 (41)	23 (36)	0.792
CD8+ T cells	34 (40)	28 (44)	0.570

Data are expressed as median and interquartile range, and percentage. Mann-Whitney for continuous and chi-square for categorical variables were performed for statistical differences between variables.

### At inclusion

**Figure 2:**
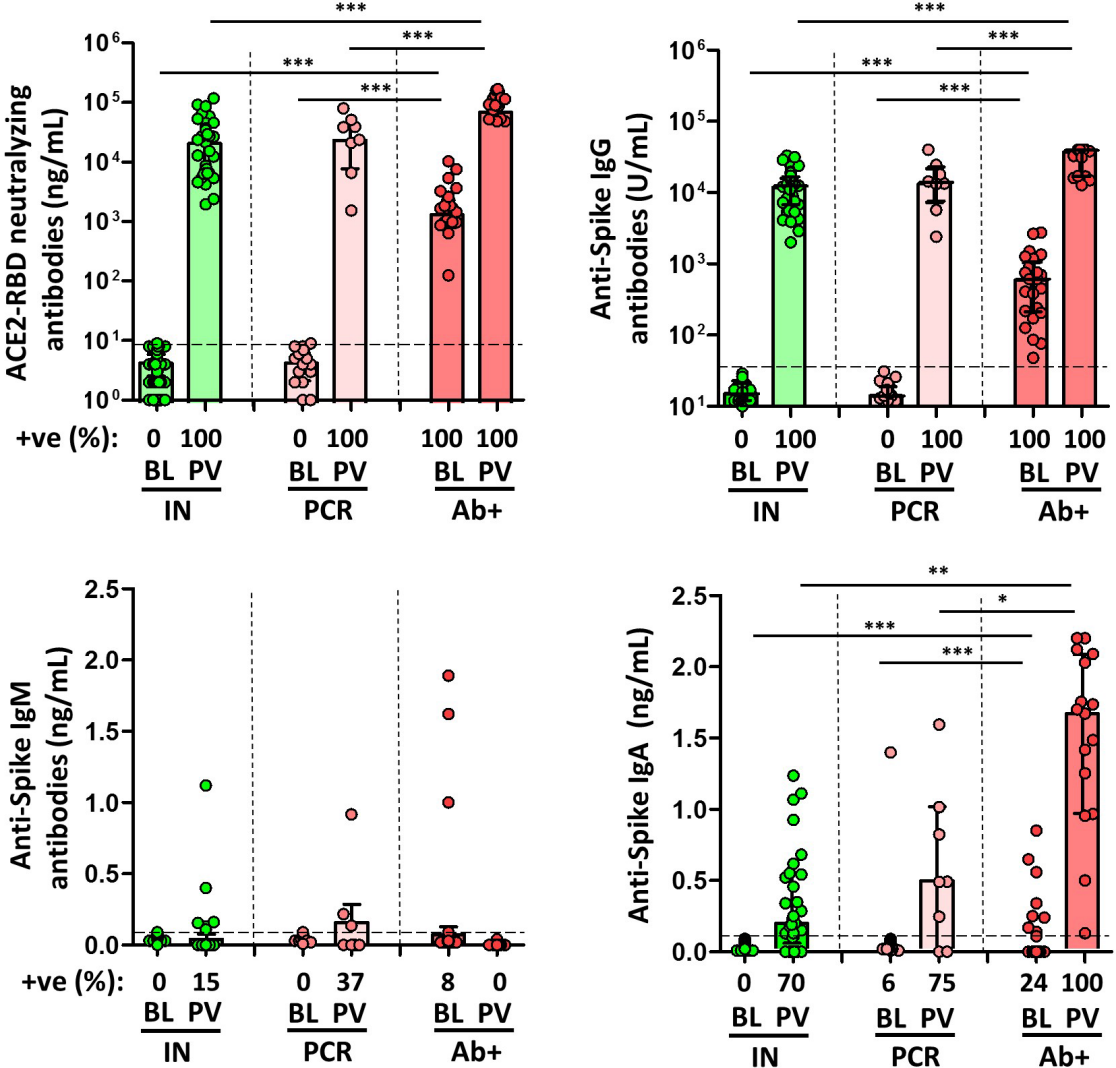
Magnitude and frequency of plasma spike-specific neutralizing, IgG, IgM and IgA antibody responses at baseline (BL) and post vaccination (PV), in infection-naïve (IN, green), and convalescent individuals (subdivided in PCR, pale, and Ab+, red, according to their initial serologic response). Only significant differences are shown. Levels of significance: *p<0.05, **p<0.01, and ***p<0.001.

SARS-CoV-2-naïve individuals were negative for specific IgG antibodies to either full-length spike protein or N protein, confirming the absence of previous infection. As shown in **Figure 2**, infection-naïve individuals had significantly lower neutralising antibodies, anti-S IgG, and IgA humoral responses compared to convalescents with detectable antibodies.

**Figure 3:**
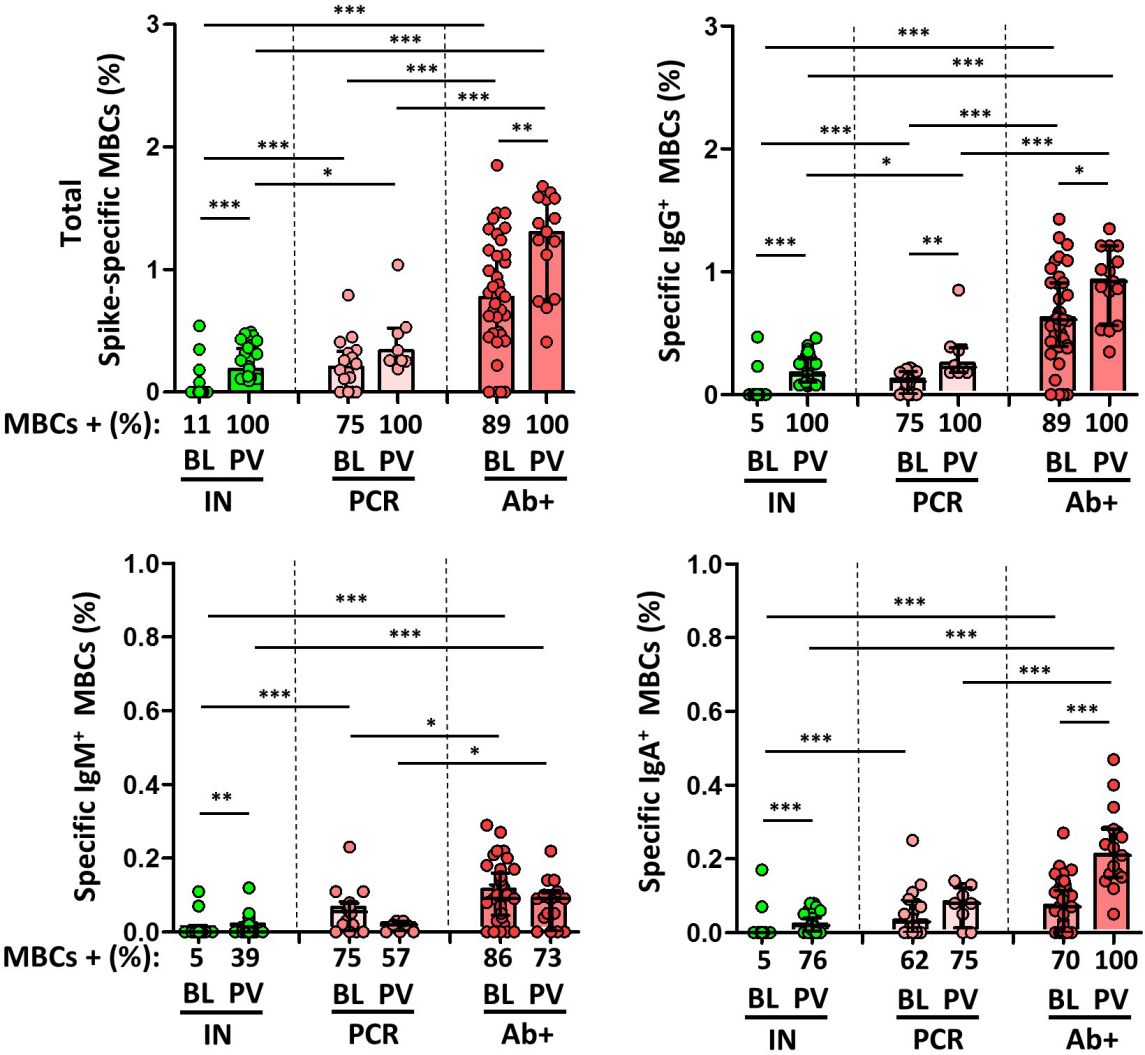
Magnitude and frequency of total specific memory B cells (MBCs), and isotypes IgG+, IgM+ and IgA+ MBCs at baseline (BL) and post vaccination (PV), in infection-naïve (IN, green), and convalescent individuals (subdivided in PCR, pale, and Ab+, red, according to their initial serologic response). Only significant differences are shown. Levels of significance: *p<0.05, **p<0.01, and ***p<0.001.

Total spike-specific MBCs were lower in infection-naïve individuals (11% with specific MBCs) compared to convalescent individuals (over 75% with specific MBCs) either with or without prior serology (p<0.0001 in both cases), as shown in **Figure 3**. In addition, IgG^+^ MBCs were found in 89% and 75% of convalescents with or without positive serology, respectively.

**Figure 4:**
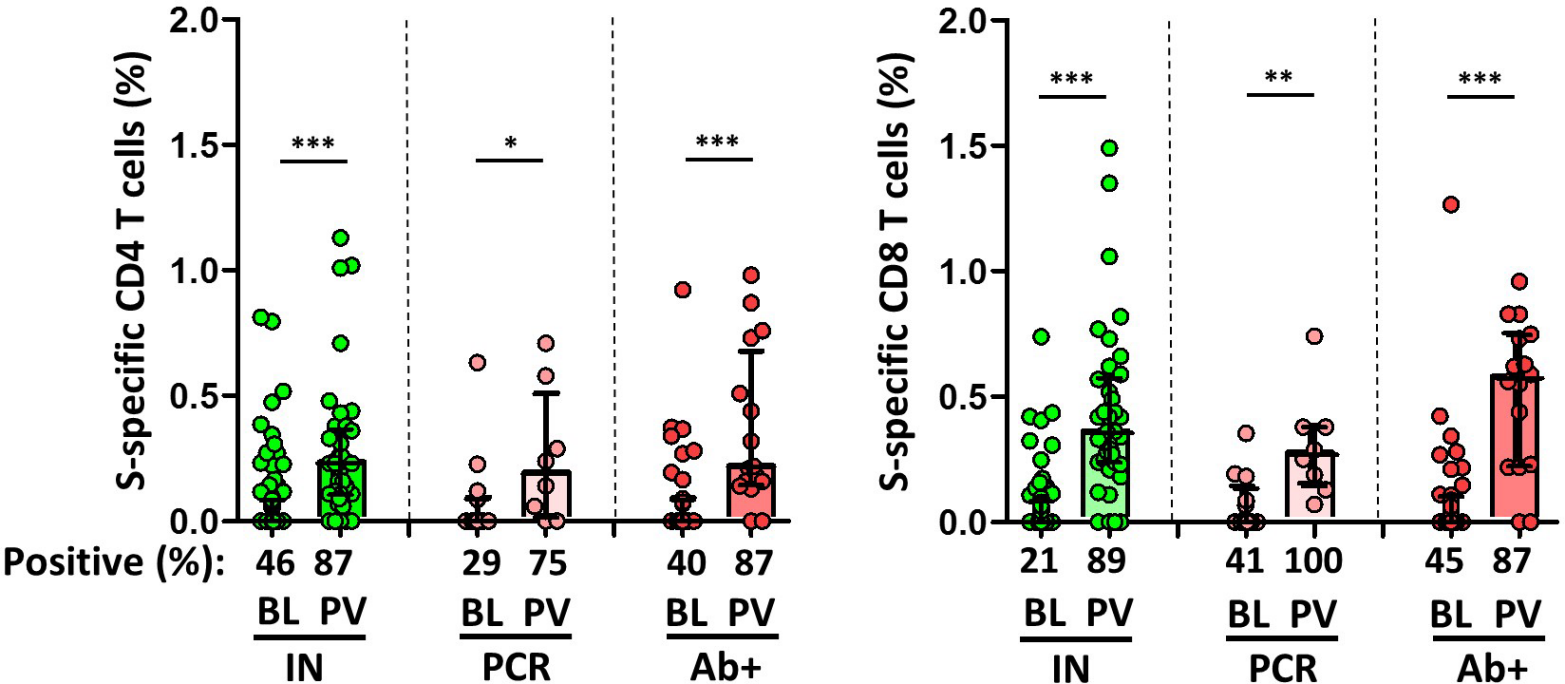
Magnitude and frequency of Spike-specific T cells at baseline (BL) and post vaccination (PV), in infection-naïve (IN, green), and convalescent individuals (subdivided in PCR, pale, and Ab+, red, according to their initial serologic response). Only significant differences are shown. Levels of significance: *p<0.05, **p<0.01, and ***p<0.001.

Of note, approximately 40% of naïve individuals without humoral response in two consecutive controls had a T cell immune response against spike protein, probably due to a cross-reactive response to common coronavirus, but lower compared to those convalescents with no previous serological response (p=0.027 for CD8^+^ T cell; **Figure 4**).

Neutralising antibodies correlated positively with anti-S IgG (r=0.565; p=0.007) and anti-S IgA antibodies (r=0.481; p=0.039) in convalescents, as shown in **Figure 4**. IgG^+^ MBCs also correlated positively with anti-S IgG antibodies (r=0.730; p=0.008), neutralising antibodies (r=0.521; p=0.009), and anti-S CD4^+^ T cells (r=0.310; p=0.019).

### Immune responses after SARS-CoV-2 mRNA vaccination

The median time from the inclusion in the study to the time after the second dose of the mRNA-based vaccine was 21 weeks (IQR, 19-23). After vaccination, all types of antibodies, except for IgM antibodies, increased significantly in all subjects. As expected, anti-S and neutralising antibody levels were significantly higher in SARS-CoV-2-experienced individuals (except for IgM antibodies), and among these, the levels were higher in those individuals with previous positive serology compared with those individuals without previous specific antibodies. Infection-naïve individuals had adequate specific IgG and IgA and neutralising antibodies, with humoral response in all individuals, as shown in **Figure 2**.

However, although spike-specific MBCs were also significantly boosted in all individuals following the administration of the second dose of vaccine, infection-naïve individuals had a significantly lower level of B cell response than that found in non-vaccinated convalescents (fluctuating between 0.9% and 0.49% of cells), and far from the levels found in convalescents (p<0.001), as shown in **Figure 3**. We also examined isotype switching in MBC in naïve and convalescent individuals after two doses of vaccine. At baseline, in convalescents, 85%, 83%, and 68% of the participants had MBCs expressing IgG, IgM, or IgA, respectively, whereas 100%, 68% and 91% of the participants had these isotypes, respectively, after vaccination. On the other hand, post-vaccination IgG^+^, IgM^+^, and IgA^+^ MBCs were present in 100%, 39%, and 76% of the infection-naïve individuals, respectively. In any case, the degree of response was significantly lower for all the isotypes compared to convalescent participants, both before and after vaccination.

**Figure 5:**
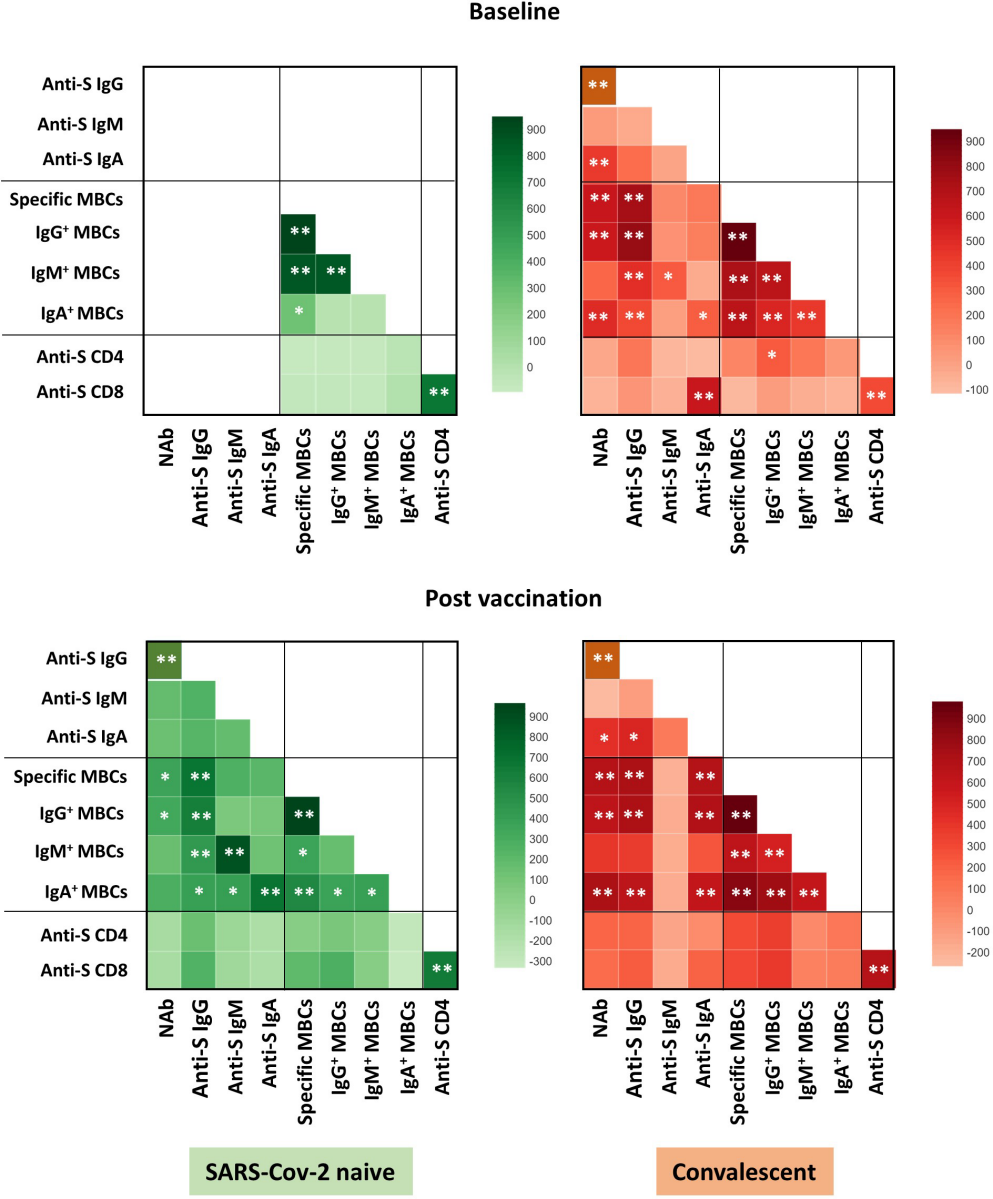
Correlation heat map of the humoral and cellular variables studied in infection-naïve and recovered individuals at baseline and post vaccination. Only significant correlations are shown. The intensity of the color indicates the R^2^ coefficient using Spearman test. Levels of significance: *p<0.05, **p<0.01.

Overall, the specific CD4^+^ and CD8^+^ T cell response increased significantly after vaccination in both infection-naïve and convalescents, but was lower in unexposed participants, as shown in **Figure 4**. Again, there was a significant correlation between spike-specific MBCs and neutralising antibodies, anti-S IgG titers, and specific CD4^+^ and CD8^+^ T cell responses in convalescents, but it is noteworthy that, this correlation remains significant between neutralising antibodies and specific MBCs, and it was not observed for T cell responses in infection-naïve individuals (**Figure 5**).

It is noteworthy to note that although the memory B cell response tended to be higher in recovered patients with severe disease compared to those with moderate/asymptomatic disease, this did not reach statistical significance (p=0.075, N=9). It is likely that the small number of patients with severe disease reduced the statistical power. In addition, both CD4 and CD8 T cell memory responses were similar between these two groups of patients (p=0.576 and p=0.455, respectively). On the other hand, age did not correlate with antibody or B/T cell responses in naïve or convalescents.

### Breakthrough infections after one year after vaccination

**Figure 6:**
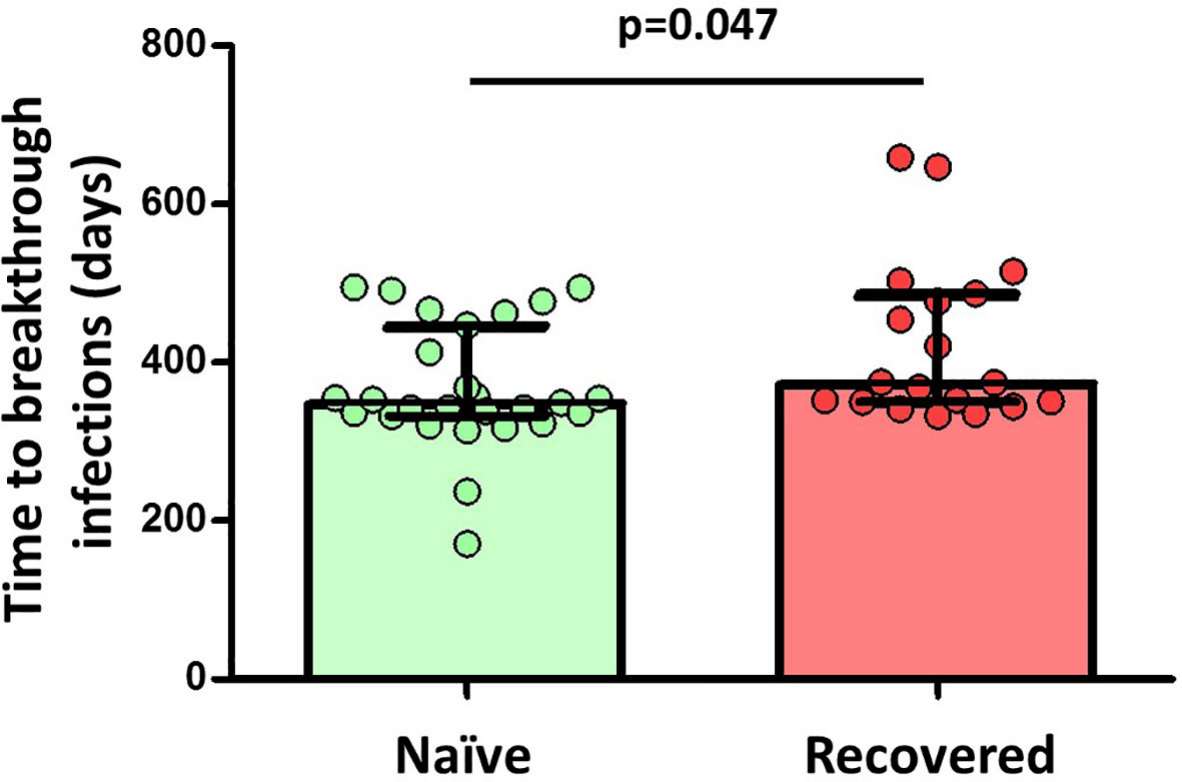
Differences in the time to breakthrough infections between naïve (29 out of 85 participants, 34%) and convalescent (20 out of 64 participants, 31%) participants among 149 participants. Significant when p<0.05.

**Figure 7:**
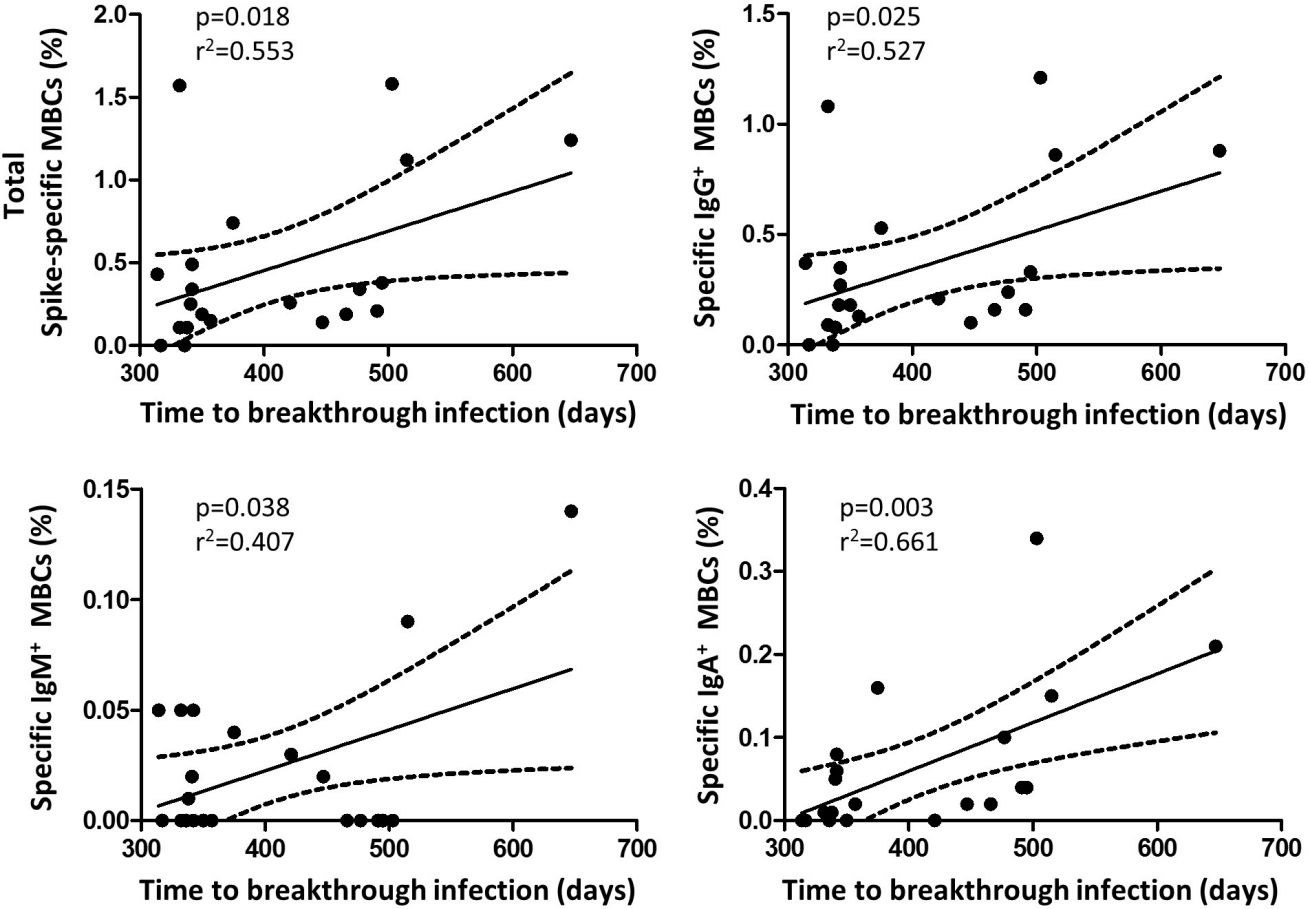
Correlation between the time of breakthrough infections with specific memory B cells and isotypes (N=21 infected participants). Significant when p<0.05.

Finally, we observed a significant inverse correlation between specific MBCs (r=-0.307; p=0.027), especially for the isotypes IgA^+^ MBCs (r=-0.279, p=0.045), neutralising antibodies, and anti-S IgG and IgA antibodies with the time from the second dose of vaccine, despite the short time between vaccine administration and the study analysis. Furthermore, during a median follow-up of 434 days (IQR, 339-495) after the two doses of the vaccine regimen, 49 individuals (33%) became infected without requiring hospitalization, with no differences in the number of naive and recovered individuals (N=28, 33% vs N=19, 30%, respectively; p=0.624; **Figure 6**). Interestingly, there was a significant direct correlation between spike-specific MBC levels after vaccination and the time of SARS-CoV-2 infection (r=0.55; p=0.018), especially for isotypes IgA^+^ and IgG^+^ MBCs (r=0.661; p=0.003 and r=0.527; p=0.025, respectively; **Figure 7**).

## Discussion

An understanding of the immune response to SARS-CoV-2 is required to evaluate long-term immunity in infection-naïve and recovered SARS-CoV-2 individuals and to develop vaccine strategies. To date, we know that specific antibodies against SARS-CoV-2 wane after a few months (3, 11, 12) due to the reduction of short-lived plasma cells. Although this has been taken as a sign of loss of immune protection, a pool of cells including MBC and memory T cells are key cells for establishing the duration of protective immune memory (13).

In this longitudinal study, we reported that specific MBCs were effectively primed by mRNA-based vaccination in most individuals, but we found that SARS-CoV-2 naïve individuals achieved significantly lower levels of specific MBCs after vaccination compared to recovered individuals. In addition, we describe for the first time that lower level of specific MBCs was associated with a shorter time to breakthrough SARS-CoV-2 infection.

Consistent with this, although several studies have shown a long duration of specific MBCs for up to 6-8 months after inclusion (7, 10, 13, 14), these studies were not designed to directly address protection or true vaccine efficacy. Indeed, previous data suggest that infection with most coronaviruses does not efficiently generate long-lived plasma cells (15, 16). A study of SARS-CoV-2-infected patients 6 years after infection showed that none of the 23 patients included had detectable specific memory B cells in ELISPOT experiments (17).

In addition, infection-induced MBCs had a better antigen-binding capacity and generated secondary MBCs, underwent more affinity maturation, and produced more robust secondary responses than vaccine-induced primary MBCs, a fact that could explain the differences in the clinical protection observed along the successive waves of SARS-CoV-2 in a well-controlled cohort of health care workers (18). Importantly, the significance of this difference in terms of immunity to subsequent SARS-CoV-2 infection could be of great importance in preventing infection with both the original and SARS-CoV-2 variants (19).

These differences in the MBC response between unexposed and convalescent individuals have been demonstrated in other studies. It has been suggested that the secondary MBCs in SARS-CoV-2- naïve individuals undergo marked affinity maturation after the second vaccine dose and that this process can continue for weeks (20). However, in contrast to the vaccine-induced primary MBCs, SARS-CoV-2-induced primary MBCs had already undergone maximal affinity maturation prior to vaccination, allowing time for maximal accumulation of somatic mutations, or because SARS-CoV-2 infection is a stronger stimulus for affinity maturation than a single mRNA vaccination (7, 21). In addition, it has been postulated that repeated antigenic stimulation may reduce the diversity of the overall response, with drifted epitopes being less targeted, explaining the differences in response (21). Thus, it might be expected that vaccinated individuals could further improve the affinity and diversity of their MBC response over time through the persistence of vaccine-induced germinal centers (22). However, our clinical data showed that MBC response was not completely able to prevent new infections, but contributed to delaying the onset of infection and probably reduced the severity of the disease (23).

Notably, we analysed the immune response in cases without a serological response after a well-documented SARS-CoV-2 infection (suggestive symptoms and confirmed positive PCR). Previously, we and others (12, 24) described a weak T cell response in those individuals with the early antibodies loss, and this development was attributed to a higher frequency of natural killer cells and an early and transient increase in specific immunoglobulin A, highlighting the role of the innate response (25). We also confirmed a higher level of IgA at inclusion, but there was no predominance of IgA+ MBCs in the convalescent individuals without a serological response. Also, a subset of pre-existing memory T cells that cross-recognized SARS-CoV-2 and different sequences of human coronavirus at single epitopes might be able to prevent infection, and these T cells could precede antibody induction after mRNA vaccination (26, 27). However, again, we did not find a higher frequency or magnitude of CD4+ and CD8+ T cells in this subset of convalescents to fully explain these differences.

We also found different MBC isotype responses after vaccination, with fewer IgM^+^ than IgA^+^ MBCs in the infection-naïve individuals. This lower IgM^+^ MBC population was also observed in seropositive convalescent COVID-19 individuals compared to IgG^+^ or IgA^+^ MBC populations, which could be explained by an increased short-term switching of MBCs. It has also been described that IgM+ MBCs contribute less to secondary antibody responses (28), and we have shown that IgA^+^ MBCs represent a lower proportion in naïve compared to convalescent individuals (10). This may be important as they play a critical role at mucosal surfaces (29) and may explain the association with breakthrough infections. In agreement with others (30), we identified specific MBCs associated with specific CD4^+^ T cells, suggesting a partial convergent development of humoral and cellular adaptive immunity in our study. Also, no association of the immune responses with disease severity was found in the convalescent group. Therefore, although other authors reported a greater memory B and T cell responses (31, 32), we could not assume this association in our cohort of patients. Furthermore, age had no effect on immune responses after vaccination (7).

We recognize the limitations of this study. First, only individuals with a recent two-dose vaccination schedule were studied; therefore, investigation of specific MBCs in patients without SARS-CoV-2 infection should be considered in subsequent studies to evaluate longer follow-up periods to decipher potential intra- and inter-variability. Second, the study was limited to the analysis after the second vaccine dose and was not extended to the third vaccine dose, especially the role of new infections with different variants of concern. Indeed, we observed breakthrough infections both with a predominance of both Delta (in 2021) and Omicron (in 2022) variants in our setting. The current global Omicron variant was not included in the experimental studies used to measure antibodies, neutralising antibodies, T and B cell responses. Finally, like most studies of adaptive immune responses in humans, our analysis was limited to the peripheral blood, although specific MBCs are likely to be found in secondary lymphoid organs.

In conclusion, our study demonstrated considerable immune heterogeneity in the generation of protective immune response, as naïve SARS-CoV-2 individuals had a shorter and weaker spike-specific MBC response both in frequency and magnitude, as compared with convalescents. Although even low levels of MBCs could prevent severe disease, these individuals may be the least protected in terms of avoiding incident infections. Other clinical implications of these findings are that two doses of vaccine should not be considered adequate doses and that good candidates with clinical complications, such as immunosuppressed individuals, should be identified to receive additional doses of vaccine with immune-boosting strategies to prevent future incident SARS-CoV-2 infections and avoid severe disease.

## Data availability statement

The raw data supporting the conclusions of this article will be made available by the authors, without undue reservation.

## Ethics statement

The studies involving human participants were reviewed and approved by Ramon y Cajal University Hospital Ethics Committee. The patients/participants provided their written informed consent to participate in this study.

## Author contributions

JC and AV conceived and designed the study, analysed the results, and wrote the manuscript. AM-H, JP, SM-C, and AV conducted the study, followed up participants, collected data, and performed analytical determinations. PV, SG-M, MF-E, and JH conducted the study, collected data, and analysed the results. All the authors revised the manuscript and approved the final version.
